# Evaluation of PLCζ and PAWP Expression in Globozoospermic Individuals 

**DOI:** 10.22074/cellj.2016.4572

**Published:** 2016-08-24

**Authors:** Majid Kamali-Dolat Abadi, Marziyeh Tavalaee, Abdolhossein Shahverdi, Mohammad Hossein Nasr-Esfahani

**Affiliations:** 1 Department of Reproductive Biotechnology, Reproductive Biomedicine Research Center, Royan Institute for Biotechnology, ACECR, Isfahan, Iran; 2 Department of Molecular and Cellular Biology, Faculty of Basic Sciences and Advanced Technologies in Biology, University of Science and Culture, Tehran, Iran; 3 Department of Embryology, Reproductive Biomedicine Research Center, Royan Institute for Reproductive Biomedicine, ACECR, Tehran, Iran; 4 Isfahan Fertility and Infertility Center, Isfahan, Iran

**Keywords:** Globozoospermia, PLCζ, PAWP

## Abstract

**Objective:**

Globozoospermia is a rare type of teratozoospermia with incidence of 0.1% among infertile individuals. Phospholipase C zeta (PLCζ) and postacrosomal sheath WW domain binding protein (PAWP) are the main candidates in sperm taking responsibility for oocyte activation during fertilization. Therefore, we aimed to evaluate the expression of these two genes at RNA and protein levels in globozoospermic individuals and compare the results with fertile individuals.

**Materials and Methods:**

In this experimental study, semen samples of 21 infertile men with globozoospermia and 25 fertile men were collected. Expression of PLCζ and PAWP at RNA and protein levels were assessed and compared between two groups by quantitative real time polymerase chain reaction (qPCR) and Western blot, respectively.

**Results:**

Expression of both PLCζ and PAWP were significantly reduced at RNA and protein levels in infertile men with globozoospermia compared to fertile men.

**Conclusion:**

This is the first study that simultaneously assessing the respective factors in a large population of globozoospermia, suggested that intra-cytoplasmic sperm injection (ICSI) along with artificial oocyte activation may rescue failed fertilization in routine ICSI.

## Introduction

With regards to high fertilization rate (around 70-80%) obtained by intra-cytoplasmic sperm injection (ICSI), this procedure remains at the forefront of assisted reproductive techniques for treating male infertility (1). Despite data highlighting remarkable application of ICSI, this technique remains futile in treatment of 3-5% of applicants due to totally failed or low number of fertilization (2). This phenomenon is determined in men with globozoospermia. 

Two kinds of globozoospermia are recognized in infertile men; i. Total or homologous globozoospermia, composed of round shape without acrosome in all sperms and ii. Partial or heterologous globozoospermia, consisting of some sperms with intact acrosome. Globozoospermia has been principally ascribed to individuals with sperm inability to activate oocyte (3). Therefore, to overcome this dearth, artificial oocyte activation (AOA) in conjunction with ICSI has been implemented, providing an opportunity for such couples to have children (4,5). Failure of sperm to induce oocyte activation has been mainly attributed to absence of sperm-borne oocyte-activating factor(s) (SOAFs) which is believed to be present in the post-acrosomal sheath of sperm perinuclear theca (PAS-PT) (6,7). 

So far, several factors have been proposed as the potential candidates for SOAFs, including phospholipase C zeta (PLCζ), postacrosomal sheath WW domain binding protein (PAWP), truncated form of KIT(tr-Kit) and citrate synthase (8,11). 

Among these factors, PLCζ has been well studied and gained the highest rank as the potential candidate. Previous study demonstrated that injection of PLCζ and PAWP recombinant proteins into the oocytes led to induce Ca^2+^ oscillations. There- fore, reduction or absence of these factors in the sperm could cause fertilization failure (12). Each of these proteins have been studied by two different research groups (12,13) and each group has provided evidences that the other factor may not be the potential candidate (14,15). Considering this ambiguity, we aimed in present study to evaluate the expression of these two genes at RNA and protein levels in globozoospermic individuals and compare the results with fertile individuals. 

## Materials and Methods

### Sperm sample preparation

This experimental study received the approval of Institutional Review Board of Isfahan Fertility and Infertility Center (IFIC) and Royan Institute (Iran). Semen samples were collected from men who had been referred to the IFIC. This study was performed on 21 infertile men with total globozoospermia and 25 fertile men recruited from couples participating in the embryo donation program, during the time period of February 2012 to April 2015. All individuals gave informed consent prior to participation in the study. All semen samples were collected by masturbation in to the sterile containers, following on 3-4 days sexual abstinence, and delivered to the laboratory within 45 minutes after ejaculation. Immediately, one portion of the semen was used for evaluation of sperm concentration and motility according to World Health Organization (WHO) guidelines (16). 

The remaining portion of the semen samples were washed twice in phosphate buffer saline (PBS, pH=7.4) and used for assessment of relative expression of PLCζ and PAWP at mRNA and protein levels by quantitative real time polymerase chain reaction (qPCR) and Western blot, respectively. 

Briefly, sperm concentration was defined using sperm counting chamber (Sperm Processor, India), sperm motility was assessed by computer assisted semen analysis (CASA) software and sperm morphology was carried out using Papanicolaou staining according to the WHO-2010 instruction. 

### Preparation of samples for quantitative real time polymerase chain reaction and Western

blot techniques Briefly, for protein and RNA extractions, semen samples were washed with PBS and lysed with total RNA isolation (TRI) reagent (SigmaAldrich, USA) according to the manufacturer’s protocol. In order to eliminate possible contamination of genomic DNA, RNA-containing samples were treated with DNaseI (Fermentas, USA). First strand cDNA synthesis was carried out using 1 mg of total RNA with the RevertAid First Strand cDNA Synthesis kit (TaKaRa, Japan). Subsequently, the obtained cDNA was kept in -70˚C freezer. 

### Western blotting technique

**Fig.1:**
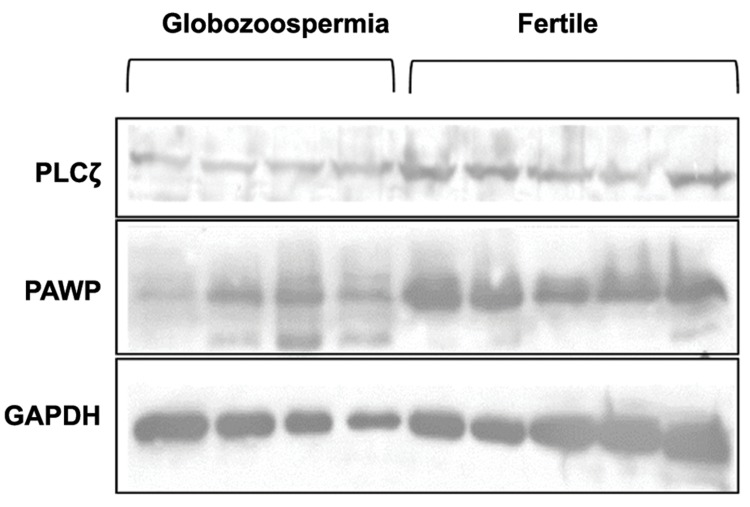
Western blot analysis of PAWP and PLCζ in 4 infertile men with globozoospermia and 5 fertile men.

Briefly, approximately 35 µg of protein was run on sodium dodecyl sulfate polyacrylamide gel electrophoresis (SDS-PAGE) and then transferred to a polyvinylidene fluoride membrane (PVDF, Biorad, USA). The membranes were blocked with skimmed milk (Merck, USA) and polyclonal antiPLCζ antibody (1:32000, Covalab, France), polyclonal anti-PAWP antibody (1:5000, abcam, UK) and monoclonal anti-glyceraldehyde-3-phosphate dehydrogenase (GAPDH), clone 6C5 (1:5000, Millipore, USA), were used as specific primary antibodies. After three times washing, the secondary antibodies, used for PAWP and PLCζ, were horseradish peroxidase (HRP)-conjugated goat anti-mouse IgG and for GAPDH was anti-rabbit IgG (all purchased from Dako, Japan). After three times washing, target proteins band were detected with an Amersham ECL Advance Western Blotting Detection Kit (GE Healthcare, Germany). The fire reader (Uvitec, UK) was used for recording chemiluminescence images. Densitometric analysis of the images was performed by Quantity One Software v 4.6.9 (Bio-Rad, Germany). Results were expressed as mean relative intensity (mean intensity of the patient’s band/mean intensity of fertile bands) (17). Figure 1 showed Western blot of PLCζ and PAWP in infertile men with globozoospermia (n=4) and fertile men (n=5). 

### Quantitative reverse transcriptase-polymerase chain reaction analysis

qPCR was carried out with a StepOnePlus Real-Time PCR System (Life Technologies, USA) as described by the manufacturer’s protocol (TaKaRa, Japan). The PCR mixture for each reaction contained 10 µl SYBR premix Ex Taq II (TaKaRa, Japan), 1 µl of each primer (5 pmol/ ml) and 50 ng cDNA adjusted to a final volume of 20 µl using dH_2_O. All reactions were carried out in triplicate. Real-time-specific primer pairs were designed by the Beacon designer 7.5 (Table 1). The real time PCR protocol composed of: 30 seconds at 95˚C followed by 40 repetitive cycles for 5 seconds at 95˚C, 10 seconds at 60˚C for *PLCζ*, and *GAPDH*, and 61˚C for *PAWP*, and then 30 seconds at 72˚C. The expression level of *PAWP* and *PLCζ* mRNA was normalized by expression of the housekeeping gene, *GAPDH*. The calculation of relative expression was assessed using the 2^-ΔΔCt^ methodas previously reported (18). 

**Table 1:** Primers used to assess PLCζ, GAPDH and PAWP mRNA levels in human sperm


Gene symbol
Primer sequences (5´-3´)

GAPDH	F: CCACTCCTCCACCTTTGACG
	R: CCACCACCCTGTTGCTGTAG
PLCζ	F: ATGCCGTTGTTTGGAGATTG
	R: AGTTTGCTTGTGAGTGTGTAG
PAWP	F: CAGATGCCTTGTTCAGTTATTGTC
	R:GCCTTCATTTCCTACGGGTTG

### Statistical analysis

For descriptive results, the data were expressed as mean ± error of mean (SE). Independent samples t test with a threshold of 0.05 was used for comparison of mean values between fertile and globozoospermic individuals. Pearson analysis was used to assess the correlations between different parameters. All statistical analyses were carried out using Statistical Package for Social Sciences (version 11.5, SPSS, Chicago, IL, USA).

### Results

The mean age of men were 40.06 ± 1.19 and 30.91 ± 1.68 years old in the fertile and globozoospermic groups, respectively. Table 2 shows the descriptive parameters including sperm concentration, percentage of motility and sperm abnormal morphology in fertile men (n=25) and individuals with globozoospermia (n=21).

**Fig.2:**
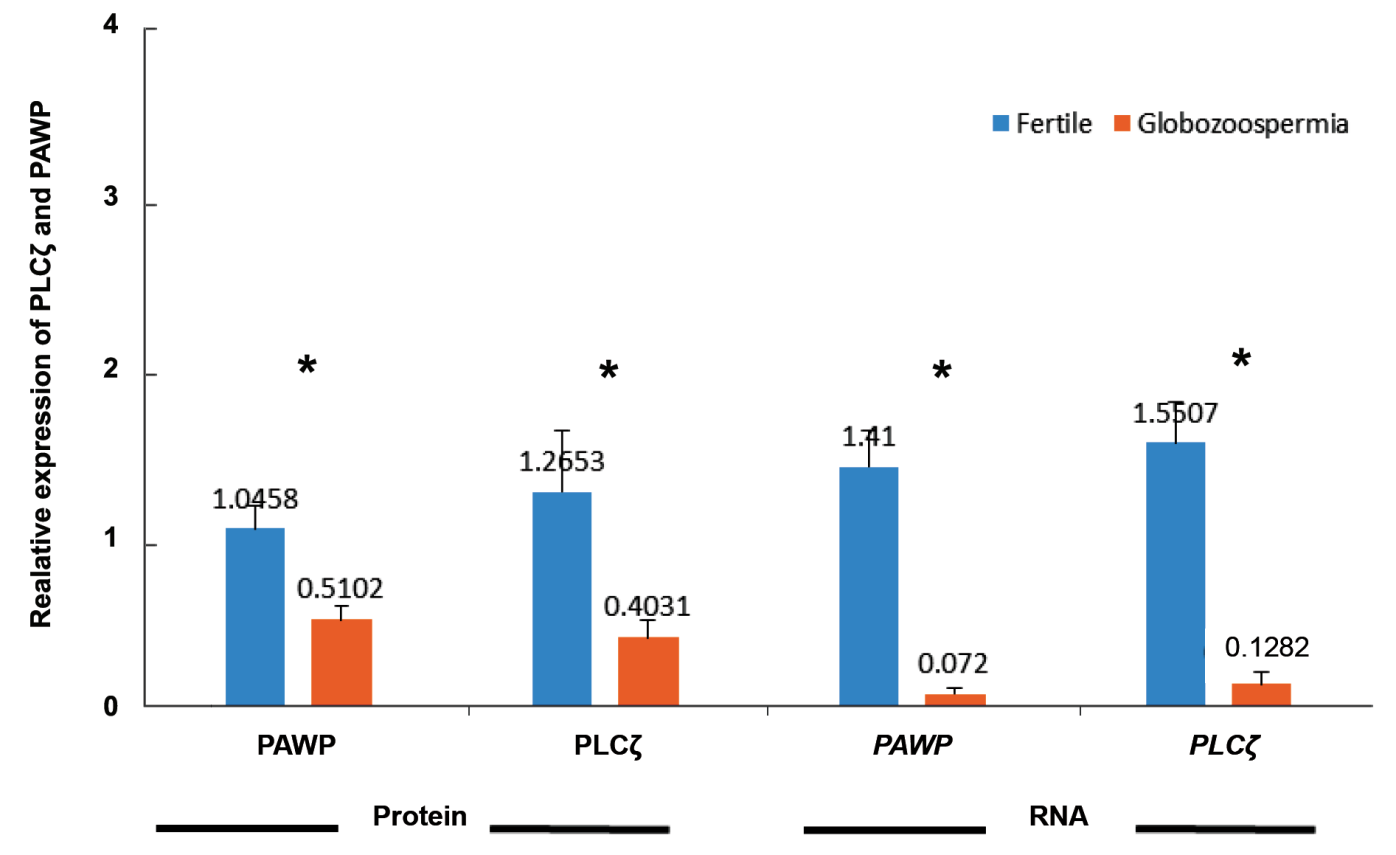
Comparison of relative expression of PLCζ and PAWP at both protein and mRNA levels between infertile individuals with globozoo- spermia and fertile men. *; Significant difference: P<0.05.

Relative expression of PAWP and PLCζ at mRNA and protein levels were compared between fertile and individuals with globozoospermia and results are presented in the Figure 2. At mRNA level, the relative expression of both PAWP (1.41 ± 0.2 vs. 0.07 ± 0.03, P<0.05) and PLCζ (1.55 ± 0.23 vs. 0.12 ± 0.07, P<0.05) were significantly lower in individuals with globozoospermia, compared to fertile individuals. Similarly, relative expression of both PAWP (1.04 ± 0.13 vs. 0.51 ± 0.08, P<0.05) and PLCζ (1.26 ± 0.36 vs. 0.40 ± 0.1, P<0.05) at protein levels were significantly lower in individuals with globozoospermia in comparison with fertile individuals.

Pearson correlation analysis between PAWP and PLCζ revealed positive significant correlations between these genes at protein level (r=0.703, P=0.000) and also at mRNA (r=0.476, P=0.003). 

**Fig.3:**
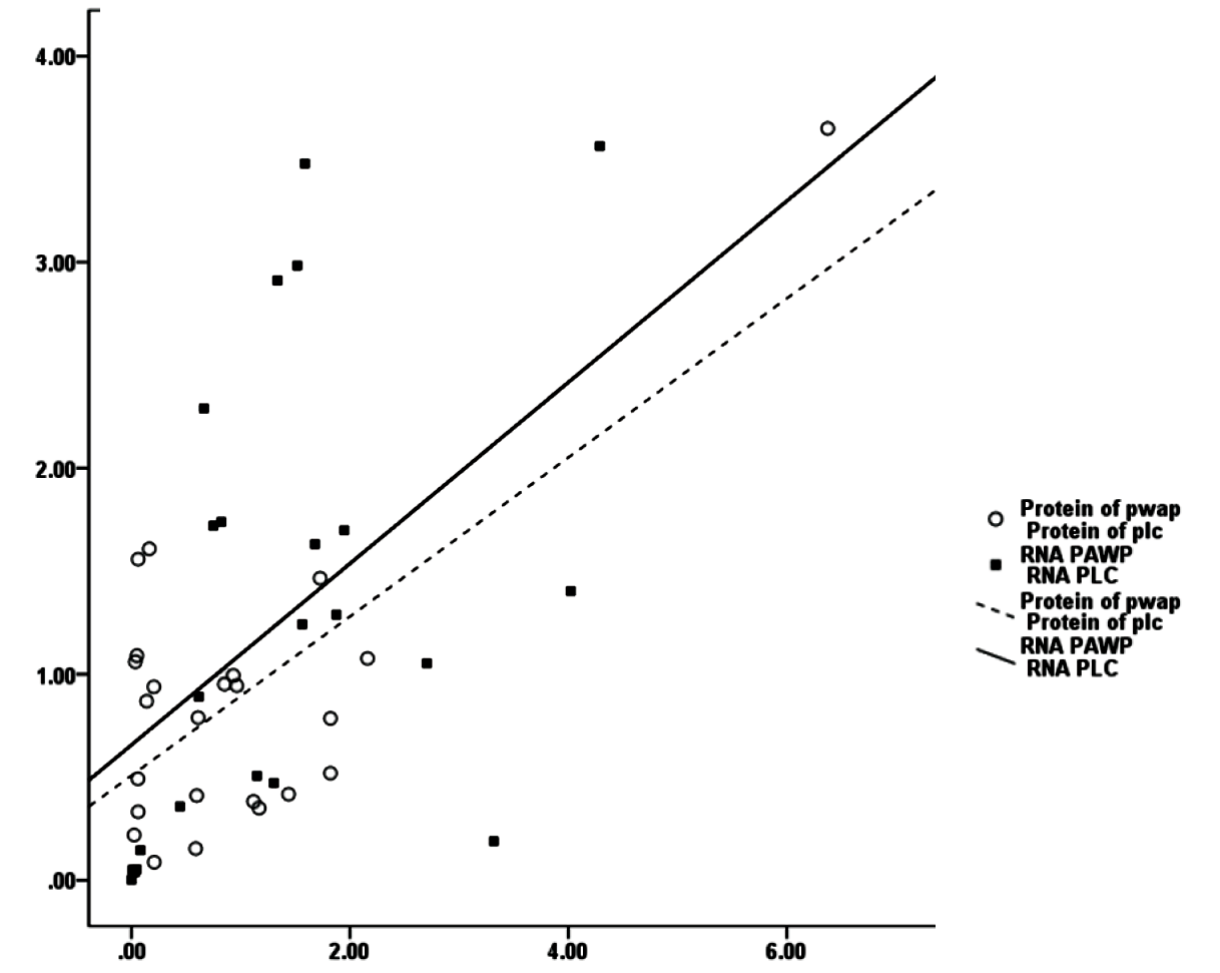
Pearson’s correlation coefficient analysis between PAWP and PLCζ at protein (r=0.703, P=0.00) and at mRNA (r=0.476, P=0.003) levels in 21 infertile men with globozoospermia and 25 fertile individuals.

However, no significant correlation was observed between the relative expression of mRNA with protein for both PAWP and PLCζ (Fig.3). 

In this study, we also assessed the correlation between semen parameters with PAWP and PLCζ at both the protein and RNA levels (Table 3). Only a significant correlation was observed between sperm morphology with the relative expression of both PAWP (r=-0.367, P=0.022) and PLCζ proteins (r=-0.375, P=0.045). At mRNA level, only *PLCζ* showed significant correlations with all the three semen parameters. 

**Table 2:** Comparison of sperm concentration, percentage of motility and sperm abnormal morphology between individuals with globozoospermia and fertile men


Parameters	Fertile Mean ± SE (n=25)	Globozoospermia Mean ± SE (n=21)	P value

Sperm concentration (10 /ml)	68.82 ± 5.0	41.57 ± 6.71	0.002
Sperm motility (%)	58.86 ± 1.54	38.44 ± 5.27	0.002
Abnormal sperm morphology (%)	94.70 ± 0.49	100 ± 00	0.000


**Table 3:** Analysis of correlation between different sperm parameters with mRNA and protein expression levels of PLCζ and PAWP, in 46 globozoospermia and fertile men


Parameters	Protein		RNA	
	PLCζ	PAWP	PLCζ	PAWP

Sperm concentration (10^6^/ml)	0.194	0.316	0.378^*^	0.017
Sperm motility (%)	0.092	0.194	0.371^*^	0.310
Abnormal sperm morphology (%)	-0.375^*^	-0.367^*^	-0.387^*^	-0.349

*; Significant difference: P<0.05.

## Discussion

Before the advent of ICSI, failure of fertilization in globozoospermia was mainly attributed to lack of acrosome and inability of such sperm to penetrate into the oocyte. However, with implementation of ICSI, it became evident that in a considerable portion of globozoospermic cases, sperm do not have the potential of inducing oocyte activation with exception of few reports achieving fertilization and pregnancy following ICSI in globozoospermic individuals (19-21).

Further research and advances in the field of reproductive medicine and animal biotechnology revealed that an essential step in the process of fertilization is the ability of sperm to induce oocyte activation. In this context, several factors have been considered as the potential candidates for SOAF, including PLCζ, PAWP, tr-kit, and citrate synthase (8-11). Among these candidates PLCζ and PAWP have gained the most attentions of two different groups, scientifically arguing which factor is more likely to be the real candidate for SAOAF (12, 13).

Considering this background and the fact that the main reason of fertilization failure, in globozoospermic individuals, is failed oocyte activation (22), which can be overcome by artificial oocyte activation (23, 24), we evaluated the presence of two potential factors in globozoospermic individuals at both RNA and protein levels and compared the results with fertile individuals. Our results revealed significant reduction at both RNA and protein levels for these two factors in globozoospermic patients compared to fertile individuals, further reiterating on our previous report implicating reduced level of *PLCζ* mRNA in these patients (18) . But, to our knowledge this is the first study evaluating the degree of expression of PAWP at both RNA and protein levels and comparing the results with fertile individuals. These results are also consistent with literature report indicating that increased expression levels of PLCζ and PAWP in human sperm strongly correlated with high fertiliation rate following ICSI, and improved embryonic quality (25, 26).

Two possibilities may account for reduced levels of PLCζ and PAWP in globozoospermia: i. Anomalies in the process of acrosome biogenesis and ii. Genetic defects underlying globozoospermia (27,29). Acrosome biogenesis has been shown to be dependent on the presence and functionality of the perinuclear theca that shields the nucleus during sperm development and contributes to signaling molecules which may be essential for oocyte activation (30,32). Therefore, lack of acrosome may account for general absence of molecules that are present in the region of pronuclear theca and candidate of SOAFs may be among the factors which are absent in such sperm (28). 

Presence of high percentage of sperm with small acrosome may lead to fertilization failure after ICSI due to similar phenomenon. The latter conclusion is in line with previous literature (27). On the other hand, reduced SOAFs may be related to genetic defects, like gene deletions and mutations associated with globozoospermia. Although it is difficult to define which option is the main cause of reduced PLCζ and PAWP in globozoospermia, the former possibility is more likely, since we detected both PLCζ and PAWP in globozoospermia at RNA and protein but at low levels. 

Electron microscopic study by Singh et al. (33) showed that in globozoospermia, acrosome is present in early spermatids and develops independently from the nucleus in the cytoplasm. The questions that remain to be defined are whether proper attachment of acrosome with nucleus could have role in localizing SOAFs in the pronuclear theca and lack of this association lead to reduced expression of PLCζ and PAWP? In addition, transfer of them into sertoli cell’s cytoplasm could lead to engulfing the residual body (27,29,34,35). Similar events have been reported for three acrosomal markers, including acrosin, intra-acrosin inhibitor and purified outer acrosomal membrane (36). Another possibility is the malfunctioning of Golgi apparatus and manchette, which may have role in propelling cargo of protein during acrosome biogenesis. Thus, such anomaly may lead to reduced deposition of *PLCζ* and *PAWP* in the perinuclear theca (37). In this regard, Dam et al. (27) and Longo et al. (38) stated that "calicin, a basic protein that is almost exclusively located in the posterior part or calyx of the sperm nuclear theca, appeared to be absent in globozoospermic cells", thereby indicating that impaired development of the spermspecific skeleton may affect protein localization in globozoospermia (39,40). Such observation has been verified in mouse model of globozoospermia with *DPY19l2* deletion, the most common genetic defect associated with human globozoospermia (29). This may also describe the punctate pattern of *PLCζ* staining within the sperm head in globozoospermic individuals (20,29). 

It is notable that despite partial absence of *PLCζ* in the sperm of some globozoospermic individuals, some oocytes became activated (41). Although the oolema deformation and the subsequent rupture of the membrane caused by ICSI procedure leads to Ca^2+^ influx. This raise may not be suffi- cient to induce oocyte activation (42,43). Nevertheless, it is possible that in some cases, this Ca^2+^influx together with the low traces of PLCζ associated with acrosomal buds might be capable of supporting oocyte activation. The importance of acrosomal buds in oocyte activation was initially suggested in a globozoospermic patient with no mutations or deletions in *SPATA16* and *DPY19L2* genes (29,44) and confirmed later in non-genotyped globozoospermic patients by Kashir et al, showing that sperms exhibited an acrosomal bud could present a punctate pattern of *PLCζ* staining within the head (45). 

## Conclusion

Level of both SOAFs candidates, PLCζ and PAWP, are reduced at both RNA and protein levels in globozoospermia. Studies of literature mainly attribute this phenomenon to impaired development of the sperm-specific skeleton in these individuals Acknowledgments This study was financially supported by Royan Institute and we would like to express our gratitude to the staff of Isfahan Fertility and Infertility for their fully supports. No potential conflict of interest relevant to this article is reported. 
